# Stress, epileptiform symptoms in schizophrenia and neural information transmission

**DOI:** 10.1515/tnsci-2025-0372

**Published:** 2025-05-09

**Authors:** Jakub Simek, Petr Bob, Ondrej Pec, Jan Chladek, Jakub Hajny, Jiri Raboch

**Affiliations:** Center for Neuropsychiatric Research of Traumatic Stress, Department of Psychiatry & UHSL, 1st Faculty of Medicine, Charles University, Ke Karlovu 11, Prague, 128 00, Czech Republic; Central European Institute of Technology, Masaryk University, Brno, Czech Republic; Institute of Scientific Instruments, Academy of Sciences of the Czech Republic, Brno, Czech Republic

**Keywords:** schizophrenia, electrodermal activity, epileptiform activity, transinformation, temporal epileptic-like symptoms, stress

## Abstract

**Background:**

Several findings indicate that stress may influence epileptiform discharges manifesting in temporal-limbic areas, which may become a potential trigger of psychosis that may manifest without neurologically diagnosed epilepsy. Some findings suggest that measures assessing levels of inter-hemispheric information connection may reveal the spread of subclinical epileptiform neural activity associated with psychotic and seizure-like symptoms. Recent research also suggests that electrodermal activity (EDA), which is related to limbic activations, may allow indirect measurement of interhemispheric information transmission. These findings about the interhemispheric spread of information suggest a hypothesis that heightened spread of information between the brain hemispheres might indirectly indicate epileptiform discharges spreading between hemispheres.

**Methods:**

We have analyzed and measured EDA and also cognitive and affective epileptic-like symptoms (CPSI, complex partial seizure-like symptoms), symptoms of chronic stress (Trauma Symptoms Checklist-40, TSC-40), and psychotic symptoms in 31 schizophrenia patients and compared these data with 31 healthy controls.

**Results:**

The results indicate that in schizophrenia patients, the values of pointwise transinformation (PTI) calculated from right and left EDA time series are related to CPSI symptoms (Spearman correlation between CPSI and PTI is *R* = 0.48; *p* < 0.01) and symptoms of chronic stress (Spearman correlation between TSC-40 and PTI is *R* = 0.37, *p* < 0.05); both during mild stress conditions caused by conflicting (incongruent) Stroop task.

**Conclusion:**

The analysis indicates potentially diagnostically useful results suggesting that heightened PTI values may reflect autonomic activations that hypothetically might be linked to higher interhemispheric transmission related to spreading of epileptiform discharges between hemispheres.

## 1 Introduction

In the historical and recent schizophrenia research there is evidence that in some cases, psychotic manifestations may be related to epileptiform discharges in temporal-limbic areas, which may trigger psychotic episodes and may manifest in patients without neurologically diagnosed epilepsy [1,2,3,4,5,6,7]. These findings are in agreement with well-known clinical experience and evidence that some patients with psychosis and other mental disorders may respond positively to anticonvulsant treatment [2,3,4,5,8,9]. In this context, recent clinical findings suggest that certain subclinical epileptiform activities may generate psychotic symptoms, which cannot be successfully treated using usual antipsychotic medication [1,2,3,7,8,9]. These subclinical epileptiform activities may have various pathogenetic factors that usually occur in temporo-limbic epilepsy, which mainly include traumatic injuries, infectious diseases, hypoxia, and other factors that may influence progressive hyperexcitability of neural activities and enhanced sensitivity in the affected brain areas, which is called “kindling mechanism” [1,2,3,7,8,9]. The term “kindling” means that repeated subthreshold electrical stimulations may cause at the beginning of the stimulation just minor electrophysiological changes, but later the stimulus of the same intensity or weaker stimulus applied under conditions of higher sensitization may determine higher EEG synchronizations and epileptiform discharges [1,4,13].

Recent findings also indicate that experienced stress and traumatic events may influence this sensitization and kindling and contribute to generation of epileptiform discharges and that also repeated stressful events may cause enhanced sensitivity to other stimuli [7,8,9]. In addition, these findings show that those repeated stress stimuli might trigger recurring episodes of psychopathological mental states [10]. This process of the so-called “behavioral sensitization” (or “kindling”) might determine that brain neural networks may become progressively sensitized toward manifestations of psychopathology [11,12,13]. These stress sensitization related processes can affect limbic and temporal lobe epileptiform activities and produce various psychopathological mental states [1,2,3,4,9,10].

All these research data indicate that not just in rare cases pathological processes related to schizophrenia might be determined by epileptiform activities in temporal and limbic areas, which cannot be directly observed on scalp electroencephalogram (EEG) or behaviorally presented as neurological seizures [1,3,4,9,10]. Further evidence suggests that this epileptiform activity may cause various psychological and neurobehavioral manifestations [2,4,6,13,14]. These psychopathological manifestations related to epileptiform neural activity are mainly localized in brain structures responsible for affective, memory, and cognitive functions [2,4]. These areas of the brain mainly include hippocampus, amygdala, and other limbic and temporal regions [15].

These brain epileptiform activities emerging as cognitive and affective disturbances produce the so-called “complex partial seizure-like symptoms” [2,3,4,5]. There is evidence that these symptoms may manifest also in patients without neurologically diagnosed epilepsy, who may have various psychiatric diagnoses such as psychosis, anxiety, depression, and other mental diseases [2,3,4,5,13,14,16].

According to epileptological research these epileptiform neural activities may be isolated in certain regions or may spread to other areas, or another hemisphere via corpus callosum and increase information flow and neural synchrony reflecting the brain epileptogenicity [17,18]. In this context, these findings about the spread of epileptiform activity suggest a hypothesis that heightened spread of information between the brain hemispheres might indirectly indicate epileptiform discharges (which may not be observable using scalp EEG). This hypothesis also suggests that such an indirect marker of epileptiform discharges might be helpful to indicate anticonvulsant therapy mainly in cases not responding to standardized pharmacological treatment.

Although schizophrenia is not related to specific abnormalities on scalp EEG, it might be linked to epileptiform activities in temporal structures, which can be indirectly measured via specific changes in the sympathetic pathways [19,20]. Because currently there is no method to measure these underlying epileptiform processes in patients without EEG abnormalities, these findings suggest that epileptiform activities might be indirectly measured via their influences on the autonomic activations. One of the very sensitive measure of these autonomic changes enables measurement of “electrodermal activity” (EDA), which in the case of bilateral measures enables to detect left-right autonomic balance and spread of neural mutual information or “transinformation” [21,22,23,24], which might indirectly reflect paroxysmal discharges [25].

## 2 Participants and methods

To test the hypothesis methods to measure complex partial seizure-like symptoms, stress symptoms, psychotic symptoms, and EDA were used in 31 participants treated in the day care program at the Psychotherapeutic and Psychosomatic Clinic ESET (range 19–56, standard deviation [SD] = 9.80; 19 males and 12 females of mean age 35.23, both with predominant high-school education; mean 14.01 years of education). The participants had diagnosis of schizophrenia (with mean period of schizophrenia 13.3 years and mean number of hospitalizations 5.07). The patient’s diagnosis was based on clinical interviewing in agreement with Diagnostic and Statistical Manual 5 (DSM-5) criteria [26] and reexamined by The Mini-International Neuropsychiatric Interview (M.I.N.I.) [27]. Patients’ treatment during assessment included only antipsychotic medication. In addition to the below mentioned exclusion criteria, no additional selection criteria were applied, and the patients were investigated in a consecutive way in the sequence of their scheduled visits. Exclusion conditions included organic brain diseases, gastrointestinal diseases and other internal illnesses, heart diseases, sensory diseases, epileptic disorders, mental disability, electroconvulsive therapy, alcohol, and drug abuse.

For statistical comparisons of the results obtained from schizophrenia participants, 31 healthy participants were assessed (mean age 34.2, range of age 21–46, SD = 7.8). The group of healthy participants consisted of 16 males and 15 females who also had predominantly high school level of education. All the controls were volunteers recruited by advertising from general population. The control sample was selected from persons who did not manifest mental disorders and also met the other exclusion criteria verified by M.I.N.I. [27]. In this case the healthy control group provides appropriate comparison because the CPSI manifest continuum of symptoms also in normal population and do not occur just in cases of severe pathological conditions [2].

## 3 Psychometric measures

Symptoms of psychosis were evaluated using the “Health of the Nation Outcome Scales (HoNOS)” [28] including 12 simple scales measuring behavior, impairment, symptoms, and social functioning. The measure is based on “external evaluation” done by a psychiatrist or psychologist and includes also the self-reported measure for participants.

Temporal epileptic-like symptoms have been evaluated by “complex partial seizure-like symptoms inventory – CPSI” [2]. CPSI evaluates cognitive-affective manifestations related temporal-limbic epileptic discharges, automatisms, dissociative symptoms, and paroxysmal somatic manifestations, which may also manifest in non-epileptic individuals with mental disorders [2,3,5,6,13,14]. The questionnaire includes 35 items assessed at “6-point Likert scale” (Cronbach’s-α = 0.95; after week test-retest reliability = 0.87).

Levels of experienced traumas and stress symptoms have been measured using the TSC-40 “Trauma Symptom Checklist” [29]. The TSC-40 includes 40 self-reported items at “4-point Likert scale.” The questionnaire identifies also adult “symptomatic clusters” in its six subscales: Anxiety, depression, dissociation, sexual abuse trauma index, sexual problems, and sleep disturbances.

## 4 EDA measurement

EDA is primarily governed by limbic modulation influences and correlates with amygdala activity but also few other structures such as prefrontal cortex, the anterior cingulate cortex and the hippocampus and elicits autonomic responses that can also influence electrical activity of the skin [21,22,23,24]. In addition some findings indicate that bilateral EDA changes may enable to reflect dynamical interactions between hemispheres and also may indirectly reflect subcortical epileptiform discharges [25].

The EDA was measured at a frequency of 1,000 Hz using two (bi-lateral) Ag/AgCl 8 mm electrodes. The electrodes were filled with electroconductive paste and attached to the medial phalanges of the index and middle finger of both hands. The participant was seated comfortably in a moderately lighted room with a monitor screen positioned approximately 100 cm in front of his/her eyes. The measurement was performed using the EDA equipment (produced by “Contact Precision Instruments, UK”) at a room temperature of 23°C during rest conditions (100 s); during the congruent non-conflicting Stroop task at about 20 s (four stimuli: “green by green ink, red by red ink, blue by blue ink, yellow by yellow ink”); and during conflicting incongruent Stroop stimuli at about 20 s (“green by red ink, red by green ink, blue by yellow ink, yellow by blue ink”) presented in the computer screen. The participant was asked to respond regularly changing questions: “name the color,” “name the word,” etc. The goal of this experimental setup in the case of the incongruent stimuli was to elicit an experience of the mild cognitive conflict.

## 5 Data analysis

In the data analysis, we have used nonlinear analytical methods describing complex dynamical systems [30]. In this context, it is possible to use a measurement of the mutual interaction and information flow between subsystems, which may be calculated via coupling measures such as PTI that may be used to study dynamical properties of the brain functions and activities of the autonomic nervous system [30,31]. In this case we have applied the PTI method on the left-EDA and right-EDA “time series” measured during the abovementioned experimental conditions and used PTI algorithm included in software package Dataplore Version 2.2 (Wildau, Germany).

The software Dataplore uses the PTI algorithm according to the simple equation (for the “*i*-time steps”):

\[Y=I({\delta }\text{,}{r}\text{,}{i})={\log }_{2}\frac{{P}_{i}^{{x}_{1}{x}_{2}}({\delta },r)}{{P}_{i}^{{x}_{1}}(r){P}_{i}^{{x}_{2}}(r)},]\]

where *x*_1_ = EDA-left and *x*_2_ = EDA-right and *r* is the radius related to the *i*th point in the “phase space.” In this context, it is possible to use a measure of the mutual interaction and information flow between subsystems that may be computed in the phase space such as PTI. This coupling measure takes into account also nonlinear dependencies and can be applied to nonstationary time series [30,31]. The PTI of two observable quantities has been derived from Shannon’s information concept and is calculated from the probability densities of the observables in the phase space. This calculation may be done using empirical point densities in the neighborhood of the reconstructed trajectory and the values of the PTI depend on the selected search radius *r* and the range of PTI values provides information about changes in the coupling behavior of the interacting systems and internal dynamic coordination of the subsystems. These analytical methods may be applied to the recorded signals representing the complex couplings of the physiological subsystems (in this case, left and right sides of the EDA). For more detailed information about variables and the method, please refer previous literature [30,31].

For the statistical analysis, Mann–Whitney test, *t*-test for the independent samples, and Spearman correlations were used with the aim to find relationships among the variables, and also descriptive statistics was calculated using software package Statistica 8.0. (Tulsa, OK, USA). Because the data did not have normal distribution, we have used mainly non-parametric statistical analysis (Spearman correlations coefficients and Mann–Whitney test). The main advantage to use non-parametric analysis is its very conservative approach to outliers and leverage points, which in the case of using parametric correlations or regression analysis may create false results and increase risk of inappropriate rejection of the null hypothesis. In addition, as previous research has indicated, this statistical analysis is also appropriate for psychopathological data reflecting traumatic stress symptoms that usually do not have normal distribution.


**Ethical approval:** The research related to human use has been complied with all the relevant national regulations, institutional policies and in accordance the tenets of the Helsinki Declaration, and has been approved by the authors’ institutional review board or equivalent committee. This study received approval from ethical committee (MEDPSYJS-0619) of the University Medical Faculty.
**Informed consent:** Informed consent has been obtained from all individuals included in this study.

## 6 Results

**Figure 1:**
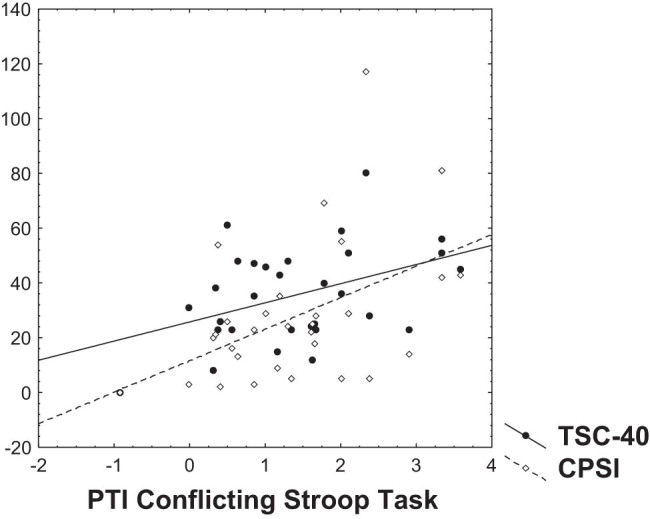
Dependency graph of the PTI (in bits – on *X*-axis) during the conflicting Stroop task with CPSI (*r* = 0.48, *p* < 0.01; on *Y*-axis) and symptoms of traumatic stress – TSC-40 (*r* = 0.37, *p* < 0.05; on *Y*-axis).

**Figure 2:**
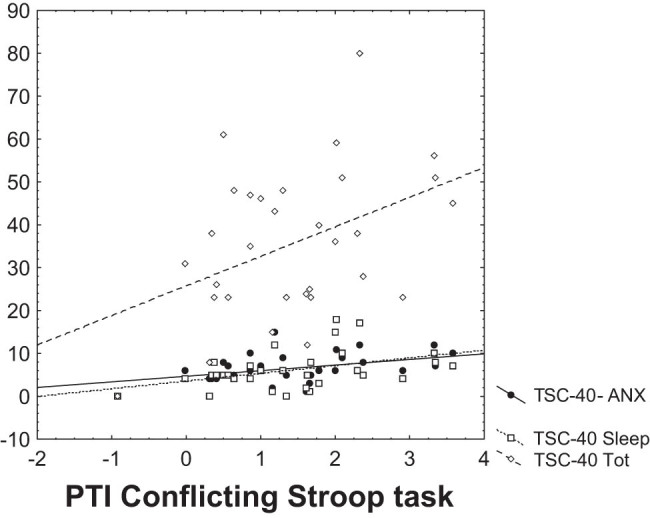
Dependency graph of the PTI (in bits – on *X*-axis) during the conflicting Stroop task with TSC-40 (Spearman *R* = 0.37, *p* < 0.05; on *Y*-axis); with TSC-40 subscale for anxiety (Spearman *R* = 0.40, *p* < 0.05; on *Y*-axis) and with TSC-40 sleep disturbances subscale (Spearman *R* = 0.45, *p* < 0.05; on *Y*-axis).

Results of the analysis and calculations indicate significant Spearman correlation during mild stress conditions related to incongruent (conflicting) Stroop task between CPSI and PTI (Spearman *R* = 0.48, *p* < 0.01) (Figure 1). Another significant Spearman correlation was found with PTI and TSC-40 (Spearman *R* = 0.37, *p* < 0.05) (Figure 1), between PTI and TSC-40 subscale for anxiety (Spearman *R* = 0.40, *p* < 0.05), and with TSC-40 sleep disturbances subscale (Spearman *R* = 0.45, *p* < 0.05) (Figure 2). Spearman correlation between PTI and HoNOS was not found. Other results of correlation analysis are given in Tables 1 and 2.

**Table 1:** Spearman correlations for PTI and psychometric measures

	PTI Rest	PTI Stroop Non	PTI Strop Con	HoNOS	TSC40- Dis	TSC-40- Anx	TSC-40 Dep	TSC-40- Sati	TSC-40- Sleep	TSC-40- Sexual	TSC-40 Tot	CPSI
PTI Rest	1.000000	0.629366	0.542603	0.303379	0.334874	0.072403	−0.073313	0.289486	0.132545	0.114995	0.159010	0.250445
PTI Stroop Non	0.629366	1.000000	0.736151	−0.061257	0.307753	0.203131	−0.011846	0.243660	0.242701	0.091996	0.181758	0.283393
PTI Strop Con	0.542603	0.736151	1.000000	0.050526	0.295425	0.406931	0.126510	0.307816	0.447339	0.257677	0.370652	0.482413
HoNOS	0.303379	−0.061257	0.050526	1.000000	0.204528	0.019201	0.138702	0.149163	0.148161	0.088747	0.246191	0.138144
TSC40- DIS	0.334874	0.307753	0.295425	0.204528	1.000000	0.588655	0.527420	0.648313	0.389127	0.356471	0.520398	0.386910
TSC-40- ANX	0.072403	0.203131	0.406931	0.019201	0.588655	1.000000	0.832286	0.714382	0.760150	0.613772	0.732080	0.614383
TSC-40 DEP	−0.073313	−0.011846	0.126510	0.138702	0.527420	0.832286	1.000000	0.752948	0.639636	0.633876	0.759917	0.442746
TSC-40- SATI	0.289486	0.243660	0.307816	0.149163	0.648313	0.714382	0.752948	1.000000	0.775367	0.773727	0.812215	0.558667
TSC-40- Sleep	0.132545	0.242701	0.447339	0.148161	0.389127	0.760150	0.639636	0.775367	1.000000	0.606407	0.632145	0.622840
TSC-40- Sexual	0.114995	0.091996	0.257677	0.088747	0.356471	0.613772	0.633876	0.773727	0.606407	1.000000	0.840068	0.638814
TSC-40 Tot	0.159010	0.181758	0.370652	0.246191	0.520398	0.732080	0.759917	0.812215	0.632145	0.840068	1.000000	0.554230
CPSI	0.250445	0.283393	0.482413	0.138144	0.386910	0.614383	0.442746	0.558667	0.622840	0.638814	0.554230	1.000000

**Table 2:** Statistical comparison of the schizophrenia patients with the group of healthy controls using *t*-test and Mann–Whitney test (*U* and *Z*)

	Schiz mean (Min;max)	Cont mean (Min;max)	*t*-test	*p*	Schiz SD	Cont SD	*U*	*Z*	*p*
PTI Rest	2.05 (−0.09;3.79)	1.64 (0.01;3.12)	1.494	0.141	1.097	1.074	367.500	1.591	0.112
PTI Stroop Non	1.436 (−0.56;3.82)	1.18 (0.01;3.42)	0.957	0.342	1.211	0.918	438.000	0.598	0.550
PTI Stroop Con	1.44 (−0.92;3.58)	1.02 (0.01;2.80)	1.789	0.079	1.055	0.758	360.000	1.696	0.090
HoNOS	12.19 (4;21)	0.00 (0.00;0.00)	16.452	0.000	4.126	0.000	0.000	6.765	0.000
TSC-40-DIS	5.48 (0;15)	2.93 (0.0;7.0)	3.211	0.002	3.880	2.112	291.500	2.661	0.008
TSC-40-ANX	6.55 (0;15)	3.68 (0.0;7.0)	3.787	0.000	3.463	2.414	249.500	3.252	0.001
TSC-40 DEP	8.65 (0;22)	5.81 (0.0;9.0)	2.830	0.006	4.910	2.664	308.500	2.422	0.015
TSC-40- SATI	6.61 (0;14)	4.26 (0.0;9.0)	3.015	0.004	3.818	2.081	305.500	2.464	0.014
TSC-40- Sleep	6.16 (0;18)	3.48 (0.0;9.0)	2.834	0.006	4.591	2.567	295.000	2.612	0.009
TSC-40-Sex.	7.29 (0;20)	4.42 (0.0;18)	2.372	0.021	5.521	3.862	334.000	2.063	0.039
TSC-40 Tot	35.68 (0;80)	15.48 (0.0;27)	5.942	0.000	17.317	7.624	137.500	4.829	0.000
CPSI	27.77 (0;117)	7.70 (0.0;18)	4.188	0.000	25.704	5.383	178.000	4.140	0.000

Additionally, as confirmatory method, we have used the Mann–Whitney test for independent samples, which indicates that the participants with CPSI higher than median have higher PTI than participants with lower CPSI (*Z* = 2.12, *p* = 0.034).

Other results obtained in schizophrenia participants did not show significant correlation of left and right EDA measured values with CPSI and other questionnaires. The same analyses in the control group of healthy participants did not show any significant correlation of CPSI and TSC-40 with the PTI.

Due to multiplicity of tests, the Bonferroni correction should be considered. It is simple application, however, requires independent tests. Looking at the pattern in Table 1, one can see that the results are not independent across questionnaires and channels, and it is hard to estimate the number of independent test groups. In order to avoid the type II errors due to taking formally all performed tests as independent, we refer the uncorrected significance. We recognize the danger of randomly occurred significant results; however, one can see in Figure 1 that the significant correlations are localized in a nonrandom pattern.

Nevertheless, in the case that we could apply the Bonferroni corrections with limitations mentioned above, then the correlation between CPSI and PTI during incongruent Stroop task (Spearman *R* = 0.48, *p* = 0.006) would remain significant (the corrected significance taken into account 4 “independent” variables TSC-40, HoNOs, CPSI, and PTI would be 0.05/4 = 0.013).

## 7 Discussion

The results show that PTI representing the information transfer between the two EDA channels (i.e., EDA transinformation) is associated with “complex partial seizure-like symptoms” and stress symptoms but not related with psychotic symptoms. Similar results reflecting this association of EDA transinformation (PTI) with “Temporal Epileptic-like Symptoms” were previously reported in a study of participants with depression [32] and in alcohol dependent patients who also may manifest reduced inhibitory functions and kindling [33]. In addition, stress and trauma symptoms assessed by TSC-40 are significantly correlated with CPSI (Table 1), which is in agreement with previously published findings that epileptiform activities may be influenced by sensitization related to chronic stress conditions [1,2,3,4,9,10].

The results suggest that increased PTI that reflects close relationship with autonomic information flow may correspond to heightened synchronization related to “hidden” epileptiform neural activities. In summary, these PTI changes may reflect increased temporal-limbic vulnerability to stressors due to repeated stressful stimuli and also neurologically “organic based” epileptic disorder [1,4,7,9,10,15,34,35] and may represent potentially applicable clinical finding suggesting that increased information transmission between hemispheres may be reflected by these measures and useful for clinical applications [7,36].

Motivation for this research is mainly based on the effort to find a clinical marker that would help to identify patients with schizophrenia who could benefit from anticonvulsant treatment. With this purpose, we are mainly focused on the hypothesis that the relationship between CPSI and PTI during mild stress condition does not mean causal relationship between these two variables. In this context, we suppose that the relationship between the CPSI and PTI represents a consequence of underlying physiological processes related to manifestation of subcortical epileptiform activity in the temporo-limbic structures that cannot be observed on scalp EEG. This hypothesis is one of many possible explanations for this effect that requires further explorations and research. Nevertheless, we emphasize this hypothesis due to its clinical significance.

The current research included in this study has few limitations and also open questions, which need to be considered in the further research. The first limitation is that of course further research needs to involve larger samples of schizophrenia patients and controls with the aim to get more general findings that could be applicable for the purpose of clinical diagnostics. Another perspective for further research will also need to elucidate more details about physiological mechanisms related to EDA that may enable to use it as an indirect measure of the neural information transmission. For this purpose, the future research could also include other methods such as EEG, mainly using intracerebral EEG, and also functional magnetic resonance imaging, which could provide useful data to understand more details about neurophysiological mechanisms underlying EDA.

Another important research perspective is to investigate the long-term effects of stress on epileptiform symptoms and related changes in neural information transmission, and to elucidate influences of various types of medication.

## 8 Conclusion

This current study is the first one examining the EDA transinformation and PTI analysis in schizophrenia patients. The results of this study indicate potentially diagnostically useful findings suggesting that elevated PTI may reflect higher neural transmission as a possible marker of epileptiform activations in patients with schizophrenia.
